# Accelerated Detection of Mycolactone Production and Response to Antibiotic Treatment in a Mouse Model of *Mycobacterium ulcerans* Disease

**DOI:** 10.1371/journal.pntd.0002618

**Published:** 2014-01-02

**Authors:** Paul J. Converse, Yalan Xing, Ki Hyun Kim, Sandeep Tyagi, Si-Yang Li, Deepak V. Almeida, Eric L. Nuermberger, Jacques H. Grosset, Yoshito Kishi

**Affiliations:** 1 Johns Hopkins University Center for Tuberculosis Research, Baltimore, Maryland, United States of America; 2 Department of Chemistry and Chemical Biology, Harvard University, Cambridge, Massachusetts, United States of America; Kwame Nkrumah University of Science and Technology (KNUST) School of Medical Sciences, Ghana

## Abstract

Diagnosis of the neglected tropical disease, Buruli ulcer, can be made by acid-fast smear microscopy, specimen culture on mycobacterial growth media, polymerase chain reaction (PCR), and/or histopathology. All have drawbacks, including non-specificity and requirements for prolonged culture at 32°C, relatively sophisticated laboratory facilities, and expertise, respectively. The causative organism, *Mycobacterium ulcerans*, produces a unique toxin, mycolactone A/B (ML) that can be detected by thin layer chromatography (TLC) or mass spectrometric analysis. Detection by the latter technique requires sophisticated facilities. TLC is relatively simple but can be complicated by the presence of other lipids in the specimen. A method using a boronate-assisted fluorogenic chemosensor in TLC can overcome this challenge by selectively detecting ML when visualized with UV light. This report describes modifications in the fluorescent TLC (F-TLC) procedure and its application to the mouse footpad model of *M. ulcerans* disease to determine the kinetics of mycolactone production and its correlation with footpad swelling and the number of colony forming units in the footpad. The response of all three parameters to treatment with the current standard regimen of rifampin (RIF) and streptomycin (STR) or a proposed oral regimen of RIF and clarithromycin (CLR) was also assessed. ML was detectable before the onset of footpad swelling when there were <10^5^ CFU per footpad. Swelling occurred when there were >10^5^ CFU per footpad. Mycolactone concentrations increased as swelling increased whereas CFU levels reached a plateau. Treatment with either RIF+STR or RIF+CLR resulted in comparable reductions of mycolactone, footpad swelling, and CFU burden. Storage in absolute ethanol appears critical to successful detection of ML in footpads and would be practical for storage of clinical samples. F-TLC may offer a new tool for confirmation of suspected clinical lesions and be more specific than smear microscopy, much faster than culture, and simpler than PCR.

## Author Summary

The diagnosis of Buruli ulcer, caused by infection with *Mycobacterium ulcerans*, is complicated by its resemblance to other diseases that may also cause ulcers in the skin. Clinical diagnosis can be supported by microscopic detection of acid-fast bacilli in the skin, by prolonged culture of at least 8 weeks, in a dedicated incubator set at 32°C, or by the polymerase chain reaction in a well-equipped laboratory usually far from the clinic where the patient comes for treatment. The treatment involves taking two drugs, one requiring injections, every day for two months, a burden for patients and their families. Since all drugs may have side effects, it is important that the treatment be appropriate for the patient's disease. We describe a new technique to rapidly and inexpensively detect the presence of the unique toxin produced by *M. ulcerans* in the mouse footpad model of Buruli ulcer. We show that the toxin can be detected in footpads before the development of signs of the disease, that more toxin is produced as the disease progresses, and that toxin levels decline in mice treated with either the current standard regimen of rifampin and streptomycin or a proposed all-oral drug regimen of rifampin and clarithromycin.

## Introduction

Buruli ulcer, a neglected tropical disease caused by *Mycobacterium ulcerans*, occurs in marshy environments in scattered countries and regions on most of the world's continents [1]. While its mode of transmission remains uncertain, it is now known that the principal virulence factor is a secreted cytotoxic lipid, mycolactone [2], whose synthetic enzymes are encoded on a giant plasmid [3]. Both features are unique among mycobacteria. Until nearly 10 years ago, the only accepted mode of treating the disease was to surgically remove the lesion and surrounding tissue followed by skin grafting [1]. Using a mouse footpad model developed in the 1950s [4] and applying the lessons of tuberculosis and leprosy chemotherapy, combination regimens of antibiotics were tested in the early 2000s [5]–[7]. The most effective treatment was found to be a combination of rifampin (RIF), an oral drug used for treatment of most mycobacterial infections, and streptomycin (STR), an injectable drug originally used to treat tuberculosis. Subsequent clinical studies [8]–[11] supported the efficacy of this regimen and resulted in the replacement of surgery with antimicrobial treatment in most programs around the world [1].

Different forms of mycolactone are produced by *M. ulcerans* in different locales. They can be detected by mass spectrometry, cytotoxicity assays, or thin-layer chromatography (TLC) [12]–[17]. The major mycolactone is mycolactone A/B [18]. Total synthesis of the mycolactones was demonstrated [19], [20] and synthetic mycolactone A/B has been made available for research purposes. Seeking to improve the TLC method by reducing background spots, Spangenberg and Kishi [17] developed a boronate-assisted fluorescent-TLC (F-TLC) method in which there is a marked reduction of background spots. The TLC plate is developed by immersion in a boronic acid acetone solution that binds to mycolactone and fluoresces on excitation by ultraviolet light. Interestingly, this method is specific to detect human mycolactones, but not fish or frog mycolactones, as well as some unknown contaminants. Unpublished results indicated that synthetic mycolactone spiked into tissue could be detected by F-TLC but detection in clinical samples was often problematic, probably due to the storage method of samples. These results prompted further development and refinement of the F-TLC assay, which is reported here.

The mouse footpad model has the advantage of a readily visible and progressive swelling of the infected foot [1], [5]–[7]. Harvested footpads can also be processed for enumeration of *M. ulcerans* colony forming units (CFU) by culture on mycobacterial media for up to 12 weeks at 32°C. Previous studies documented histological and microbiological changes after infection as well as changes in toxin levels in frozen footpads before and after treatment with RIF+STR [21]–[26]. Among the goals of the World Health Organization Global Buruli Ulcer Initiative is to find an all-oral regimen, in other words to replace STR with an oral alternative such as clarithromycin (CLR) [27]–[29].

In the current study, we documented weekly changes in footpad swelling, CFU counts, and toxin levels in infected mouse footpads before and after treatment, comparing the efficacy of RIF+STR and RIF+CLR. After preliminary studies with convenience samples, we were able to show that mycolactone may be best preserved not by freezing but by storage in absolute ethanol, a finding that could also be of practical benefit under field conditions.

## Materials and Methods

### Bacteria

*M. ulcerans* 1615 (Mu1615), an isolate originally obtained from a patient in Malaysia in the 1960s [30], was kindly provided by Dr. Pamela Small, University of Tennessee. According to Dr. Small (personal communication), this strain is a stable producer of mycolactone A/B whereas modern African strains often lose the capacity to produce mycolactone unless passaged in mice (Drs. Stewart Cole and Laurent Marsollier, personal communication to JHG). Previous studies have confirmed that this strain produces mycolactone A/B and kills macrophages and fibroblasts [24], [31], [32]. The strain was passaged in mouse footpads before use in these studies. The bacilli were harvested from swollen footpads at the grade 2 level, i.e., swelling with inflammation of the footpad [6].

### Ethics statement

All animal procedures were conducted according to relevant national and international guidelines. The study was conducted adhering to the Johns Hopkins University guidelines for animal husbandry and was approved by the Johns Hopkins Animal Care and Use Committee, protocol MO11M240. The Johns Hopkins program is in compliance with the Animal Welfare Act regulations and Public Health Service (PHS) Policy and also maintains accreditation of its program by the private Association for the Assessment and Accreditation of Laboratory Animal Care (AAALAC) International.

### Antibiotics

RIF and STR were purchased from Sigma (St. Louis, MO). CLR was kindly provided by Abbott (Abbott Park, IL). STR and RIF were dissolved in distilled water, and CLR was dissolved in distilled water with 0.05% agarose for administration to mice. All drugs were administered 5 days per week in 0.2 ml. RIF (10 mg/kg) and CLR (100 mg/kg) were administered by gavage. STR (150 mg/kg) was administered by subcutaneous injection.

### Infection and CFU analysis

BALB/c mice, age 4–6 weeks (Charles River, Wilmington, MA), were inoculated in the right hind footpad with approximately 4.54 log_10_ (3.45×10^4^) CFU of Mu1615 in 0.03 ml PBS. Footpads were harvested weekly from 8 mice (5 for CFU count, 3 for ML detection) at different time points after infection (Table 1) and before treatment, up to ≥grade 3 swelling. After the onset of grade 2 swelling (week 6), treatment with RIF+STR or CLR was administered for 5 weeks (week 11 after infection). Groups of treated mice were also sacrificed for these analyses. Footpad tissue was harvested, minced with fine scissors, suspended in 2.5 ml PBS, serially diluted, and plated on Middlebrook selective 7H11 plates (Becton-Dickinson, Sparks, MD). Plates were incubated at 32°C and colonies were counted after 10 weeks with a final determination at 12 weeks of incubation.

**Table 1:** Experimental scheme.

Week	0	1	2	3	4	5	6	6.1*	6.5†	7	8	9	10	11	Total
CFU (no R_x_)	5	5	5	5	5	5			4	5	5				44
CFU RS									5	5	5	5	5	4	29
CFU RC									5	5	5	5	5	4	29
ML (no R_x_)	3	3	3	3	3	3		3	3	3	3	3	3	3	39
ML RS								3	3	3	3	3	3	3	21
ML RC								3	3	3	3	3	3	3	21
**Total**	8	8	8	8	8	8		9	23	24	24	19	19	17	**183**

Mice were randomized after infection with 0.03 ml of an inoculum containing 6.06 log_10_/ml;

Treatment with RS: RIF, 10 mg/kg, +STR, 150 mg/kg or RC: RIF, 10 mg/kg, +CLR, 100 mg/kg.

Treatment (R_x_) start time was week 6 when mice had average swelling grade of 2 and continued for 5 weeks (i.e. week 11 after infection);

* Week 6+1 Day treatment;

† Week 6+3 Days treatment;

CFU counts done only on infected right hind footpad (RHFP);

ML = Mycolactone A/B detection experiments done on both footpads.

### Analysis of mycolactone A/B

#### Tissue harvest

Footpads were harvested for detection of mycolactone by stripping bottom and top sides of the infected and contralateral footpads and then immediately immersing the two sides into a polypropylene Micrewtube® tube with O-ring and screw cap (Simport Scientific, Beloeil, QC, Canada) containing 750 µl absolute ethanol. Preliminary experiments indicated that mycolactone is stably preserved in ethanol for at least 3 weeks. Tubes were wrapped and kept in the dark at room temperature. Samples were usually shipped overnight to the Harvard lab within 24 hours and on one occasion after 7 days for logistical reasons.

#### Tissue processing

**Figure 1:**
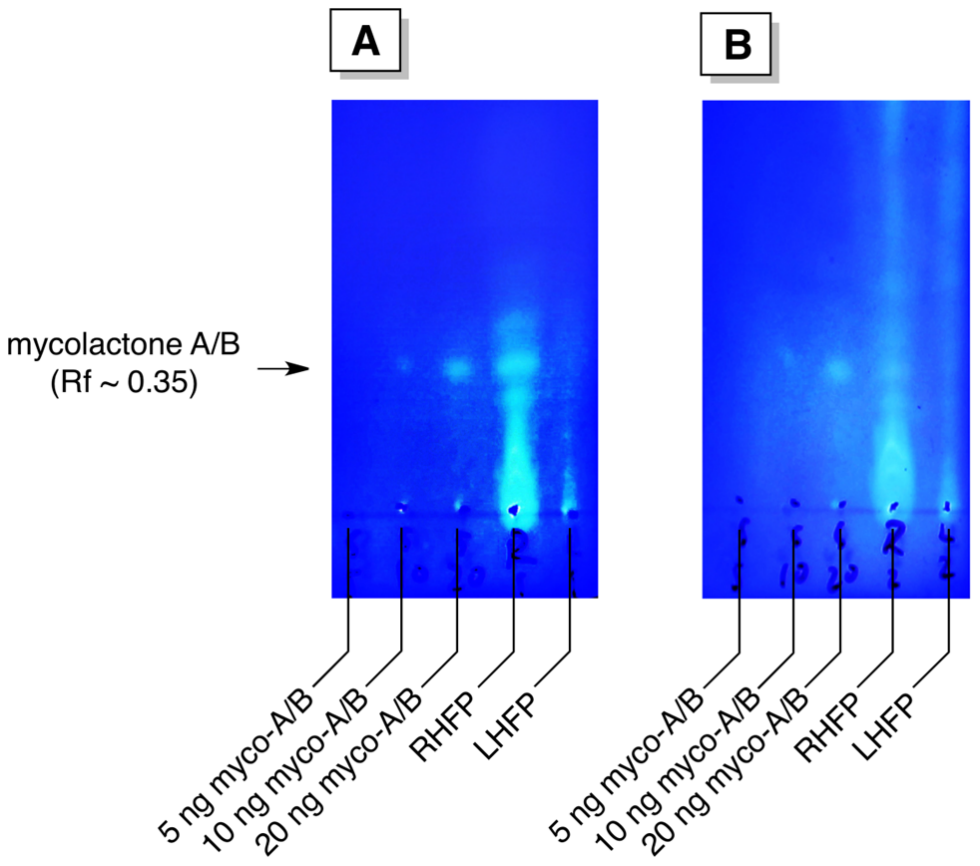
TLC-pictures for Week 8 of untreated and RS-treated mice. TLC-photos were taken and subjected to resolution enhancement (Adobe Photoshop CS 6). Panel A: untreated mouse. By visual comparison with the controls, the amount of mycolactone A/B in the right hind footpad (RHFP) sample was estimated to be 35–40 ng, which corresponds to 50–57 ng per footpad. No mycolactone A/B was detected in the left hind footpad (LHFP). Panel B: RS-treated (for 2 weeks) mouse. By visual comparison with the controls, the amount of mycolactone A/B in the RHFP sample was estimated to be 15–20 ng, which corresponds to 21–28 ng per footpad. No mycolactone A/B was detected in the LHFP.

The EtOH solvent was transferred to a glass vial (VWR 66011-041) and evaporated. Footpad was weighed (wet weights of footpads varied from ∼50 mg (grade 1 infection) up to 120 mg (grade 4 infection)), before transferring the tissue to a 7-ml Dounce tissue grinder and homogenization in 1.0 mL ethyl acetate (EtOAc). The homogenate was filtered through a Pasteur pipette containing a cotton plug into the original glass vial that had contained the EtOH solvent. The pestle was then rinsed with ∼1.5 ml EtOAc and the solvent was again evaporated. After evaporation, 50 µl of EtOAc was added to the vial and 35 µl was spotted onto a 3×6 cm fluorescent-dye free TLC plate (TLC Silica gel 60, EMD Millipore, Darmstadt, Germany; Gibbstown, NJ, USA) along with spots for 5, 10, and 20 ng synthetic mycolactone A/B standards. The plates were developed in 90∶10∶1 chloroform∶methanol∶water, air-dried, and dipped in boronic acid [17], heated for 5∼10 seconds at 100°C, before wiping the glass back with acetone on a paper towel. The plate was placed on a UV lamp with a 365 nm filter. Fluorescent spot intensity was compared to that of the standards to estimate the amount of mycolactone A/B in the sample. TLC photos were taken and subjected to resolution enhancement (Adobe Photoshop CS 6). TLC-pictures thus obtained serve for recording purposes, although the sensitivity with eye-analysis is better. For illustration, F-TLC pictures for Week 8 of untreated and RS-treated (for 2 weeks) are shown in Figure 1.

As 70% of the EtOAc solution (35 µl out of 50 µl EtOAc) was used for F-TLC analysis, an amount of mycolactone A/B present in a footpad corresponds to (estimated amount from F-TLC) ng×(50/35).

### Statistical analysis

GraphPad Prism 4 was used to compare group means by student's T test and analysis of variance and linear regression analysis for comparison of slopes and intercepts

## Results

### Footpad swelling

**Figure 2:**
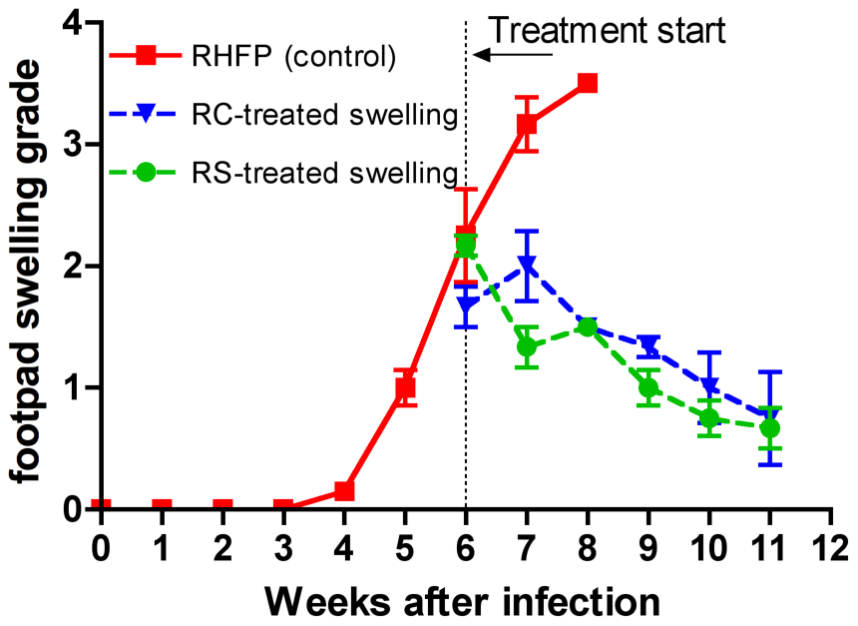
Footpad swelling in mice infected with *M. ulcerans* before and after antibiotic treatment. After infection in the right hind footpad (RHFP), grade 1 swelling was first detected at 5 weeks. Treatment began when swelling averaged grade 2 at week 6. Swelling continued to increase for the next two weeks to grade 3.5 in untreated control mice (red squares). Swelling was arrested in mice treated with either rifampin and streptomycin (RS, green circles) or rifampin and clarithromycin (RC, blue triangles) and then declined at a comparable rate to a grade of <1 over the 5-week treatment period used in this experiment. No swelling occurred in the uninfected contralateral left hind footpads.

The most rapid but least specific method of assessing *M. ulcerans* infection in human disease is to check for typical lesions. In the mouse model where infection time is known, the method is straightforward and well documented [6], [24], [27], [31]. Figure 2 shows a detailed assessment of swelling progression with weekly observations. Swelling was suggested as early as 4 weeks after infection with unambiguous enlargement (grade 1±0.25) of footpads at week 5. The average footpad-swelling grade increased to level 2 (2.25±0.66) at week 6 and increased again to grade 3 (3.17±0.38) at week 7. At week 8 when the average reached 3.5±0.00, mice were sacrificed per protocol. The contralateral uninfected footpads showed no indications of swelling throughout the experiment. What was unknown in this time course was whether swelling is preceded by the presence of detectable mycolactone or if the number of organisms present in the footpad determines swelling.

### Mycolactone production

**Figure 3:**
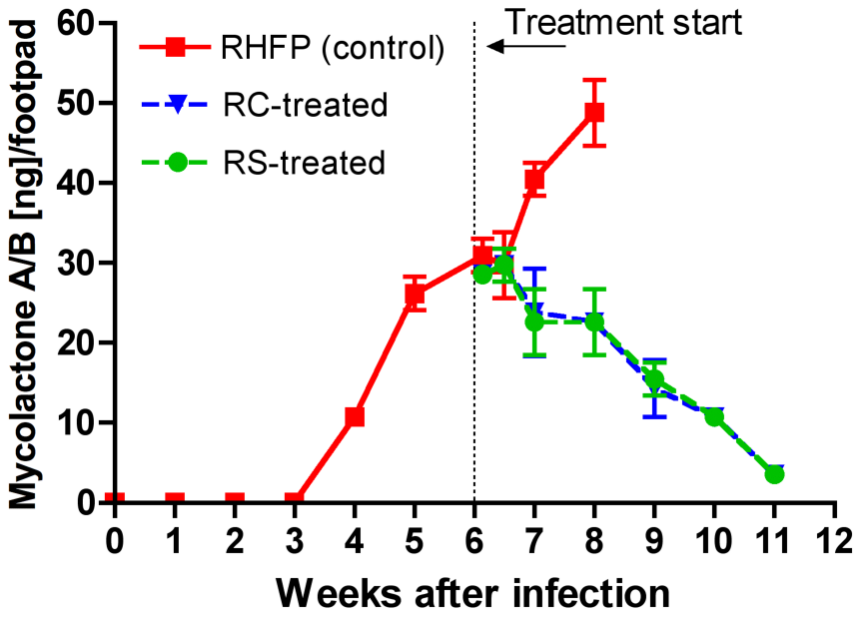
Detection of mycolactone A/B in footpads infected with *M. ulcerans* before and after antibiotic treatment. After infection with *M. ulcerans* in the right hind footpad (RHFP), ∼11 ng (∼7.5 ng×50/35) mycolactone A/B per footpad was detected at 4 weeks, one week before the observation of grade 1 swelling of the footpads. Treatment began when swelling averaged grade 2 at week 6 and mycolactone A/B levels were at 31±2 ng ((22±2 ng)×50/35) per footpad. Mycolactone production continued to increase for the next two weeks in untreated control mice (red squares), reaching 49±4 ng ((34±3) ng×50/35). Mycolactone production was arrested in mice treated with either rifampin and streptomycin (RS, green circles) or rifampin and clarithromycin (RC, blue triangles) and then declined to near undetectable levels (<5 ng) over the 5-week treatment period used in this experiment. At no time was mycolactone A/B detected in the contralateral left hind footpads.

In this experiment, footpads stored in absolute ethanol were shipped overnight to the chemistry lab and quantitative mycolactone A/B results were determined within 24–48 hours of footpad harvest. As shown in Figure 3, mycolactone was detectable at ∼11 ng (∼7.5 ng×50/35)/footpad at week 4, one week before the observation of unambiguous footpad swelling. The amount increased to 26±2 ng ((18±2 ng)×50/35)/footpad at week 5, 31±2 ng ((22±2 ng)×50/35) at week 6, the beginning of treatment, 40±2 ng ((28±3 ng)×50/35) at week 7, and 49±4 ng ((34±3 ng)×50/35) at week 8 in untreated mice. These results indicate that the mycolactone toxin is present in “pre-clinical” lesions and can be detected in footpad tissue extracts by fluorescent TLC.

### *M. ulcerans* multiplication

**Figure 4:**
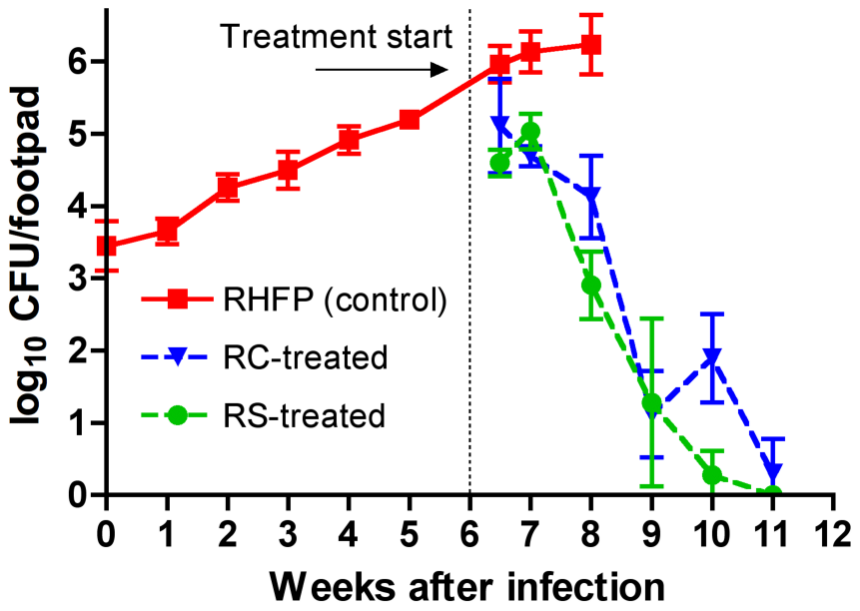
*M. ulcerans* CFU in mouse footpads before and after antibiotic treatment. After infection in the right hind footpad (RHFP), there were increases in the numbers of *M. ulcerans* detected in the footpads. At week 4, the time of detectable mycolactone A/B, there were 4.9±0.2 log_10_ CFU in the footpads and there were 5.2±0.1 log_10_ CFU at week 5, the time of observable footpad swelling. CFU levels peaked at 6.2±0.4 log_10_, at week 8 in untreated mice, little changed from the 6.0±0.3 log_10_ found at the beginning of treatment at week 6. *M. ulcerans* growth was arrested in mice treated with either rifampin and streptomycin (RS, green circles) or rifampin and clarithromycin (RC, blue triangles). After 5 weeks of RS treatment, all footpads were culture negative whereas 3/5 mice treated with RC were culture negative.

Using quantitative culture at 32°C on selective Middlebrook 7H11 plates, countable colonies were present only after 10 weeks. At baseline, on day 1 after infection the CFU counts were 3.45±0.34 log_10_ per footpad. There was a gradual increase weekly with the CFU burden being 3.65±0.17, 4.26±0.18, 4.50±0.26, 4.92±0.19, and, at the time of detectable footpad swelling, just over 5 log_10_ at 5.20±0.10 log_10_ per footpad at weeks 1, 2, 3, 4, and 5, respectively, after infection (Figure 4). After initial footpad swelling, there was a further increase in *M. ulcerans* CFU to 5.96±0.26 log_10_ at which point there was a plateau with counts at subsequent weeks (7 and 8) being 6.13±0.28 and 6.23±0.41 log_10_/footpad, respectively. However, swelling and mycolactone production continued to increase. From these data, we conclude that footpad swelling only occurs after there are approximately 5log_10_ organisms in the footpad and that bacterial multiplication increases only slightly after that time while footpad swelling increases dramatically.

### Impact of RIF+STR and RIF+CLR treatment

#### Footpad swelling

On average, footpad swelling decreased steadily after the onset of treatment (week 6) with either the current standard regimen of RIF+STR or the proposed oral regimen of RIF+CLR from an average of grade 2 to an average of less than grade 1 as shown in Figure 2. Treatment was for only 5 weeks rather than the standard 8 weeks, which we have shown previously [24] using RIF+STR to render all or nearly all mice free of detectable footpad swelling. From these data, it would appear that the RIF+CLR combination should be equally effective as the RIF+STR combination in eliminating apparent signs of disease.

#### Mycolactone production

Over the 5 weeks of treatment, there was also a steady decrease in the production of detectable mycolactone A/B by F-TLC, regardless of antimicrobial regimen. This suggests that the drugs cripple mycolactone production either by inhibiting the machinery of the *M. ulcerans* plasmid or by killing the organism. Levels declined from 29±2 ng/footpad at the beginning of treatment to less than 5 ng/footpad after 5 weeks of treatment at week 11. Again, neither regimen displayed discernible superiority on disabling the production of mycolactone A/B (Figure 3).

#### *M. ulcerans* multiplication and survival

As shown previously [24], [27], there is a rapid decrease in the number of *M. ulcerans* CFU after the beginning of treatment with RIF+STR. The decrease in this experiment was greater during the second week of treatment than during the first week, particularly in the RIF+STR-treated mice and these mice were all culture negative by the completion of 5 weeks of treatment (Figure 4). There was a parallel decrease in the RIF+CLR-treated mice in which 3 of 5 mice were culture negative by the completion of treatment. In summary, although there was no significant difference between the slopes of the curves of the two regimens as assessed by linear regression analysis, the time to culture negativity at 10.5 weeks vs. 11.1 weeks was statistically significantly earlier (p<0.025) in mice treated with RIF+STR.

## Discussion

The described developments in the fluorescent TLC procedure for the detection of mycolactone A/B in mice infected with *M. ulcerans* may have practical implications. This detection technique for the unique toxin of *M. ulcerans* may enable simpler and earlier specific diagnosis of Buruli ulcer in humans. Acid-fast microscopy for detection of *M. ulcerans* is also relatively rapid but lacks both sensitivity and specificity and histology requires expertise often not present in endemic areas. Molecular tests can also be applied but have similar limitations though PCR is relatively sensitive [33], [34]. The most sensitive and specific method of detection is culture at 32°C on microbiological media but it requires up to 8 weeks for detection and up to 12 weeks for quantification in this model [24], [33], [35] and is prone to contamination. Here, detection of mycolactone, a specific marker of *M. ulcerans*, was achieved within days of tissue harvest and even before the presence of a lesion (i.e., footpad swelling) in the mouse model. Compared to our previous studies [24] with frozen footpads and mycolactone detection by mass spectrometry, storage in absolute ethanol appears to be the key for detection at earlier phases of the infection and to increase sensitivity of TLC. Ethanol is also a more practical option than deep-freezing in the field. Experiments comparing ethanol with iso-propanol and ethyl acetate as the storage medium indicated that iso-propanol might be slightly superior to ethanol but that ethanol is clearly superior to ethyl acetate (unpublished observations). The differences were principally in the degree of diffusion of the spots. In footpads not kept in any of these solvents, there was almost complete elimination of detectable mycolactone.

We have also more precisely determined the relationship between footpad swelling, toxin production, and bacterial multiplication. Mycolactone A/B can be detected before the onset of footpad swelling when there are <10^5^ bacilli per footpad and swelling occurs when there are >10^5^ bacilli in the footpad. As observed previously [24], there is a plateau in bacillary numbers soon after the onset of swelling, even though swelling continues to increase. Unlike the CFU counts, toxin concentrations continue to increase as swelling increases. It remains to be determined how this dynamic interaction occurs in human lesions, which are not at all as circumscribed as those in the mouse. In humans, it will also be important to determine the best sites within lesions and the best techniques (e.g., swab or fine needle aspirate) to obtain toxin-containing specimens.

The impact of drug treatment with RIF-STR on footpad swelling and cultivable *M. ulcerans* was confirmed in these experiments. Importantly, we also observed a reduction in detectable mycolactone after the onset of drug treatment. In all three cases, the observations were made on a weekly basis providing further precision to the observations. We also found that the all-oral alternative RIF+CLR regimen for Buruli ulcer treatment, though possibly less bactericidal, appears to be equally active as the standard RIF+STR regimen in reducing swelling and blocking toxin production. Thus, the inhibition of the enzymatic machinery involved in producing a virulence factor may be as effective as and possibly less toxic than the killing of the bacteria.

The fluorescent TLC method could be an excellent tool for both the rapid and early detection of *M. ulcerans* infection and for monitoring the response to antimicrobial chemotherapy. The assay is practical in that absolute ethanol is readily available in all clinics and mycolactone is preserved in absolute EtOH at room temperature for at least 3 weeks (data not shown). The assay should be practical in intermediate level laboratories, thus facilitating confirmation of diagnoses before the onset of ulceration or soon after the onset of treatment.

## Supporting Information

Figure S1 **Schematic of fluorescent thin layer chromatography (f-TLC) procedure with examples.** Top left) Explanation of the f-TLC method; Top right) Schematic of f-TLC layout. Center, Stained TLC plates of mouse footpads, untreated on left and treated (RS) on right, from top to bottom: 6, 7, and 8 weeks after infection.
(TIF)
