# Supplementary figures and images for: Accelerated Detection of Mycolactone Production and Response to Antibiotic Treatment in a Mouse Model of Mycobacterium ulcerans Disease
Source: PLoS Negl Trop Dis. 2014 Jan 2;8(1):e2618. doi: 10.1371/journal.pntd.0002618 (PMC3879254; doi:10.1371/journal.pntd.0002618)

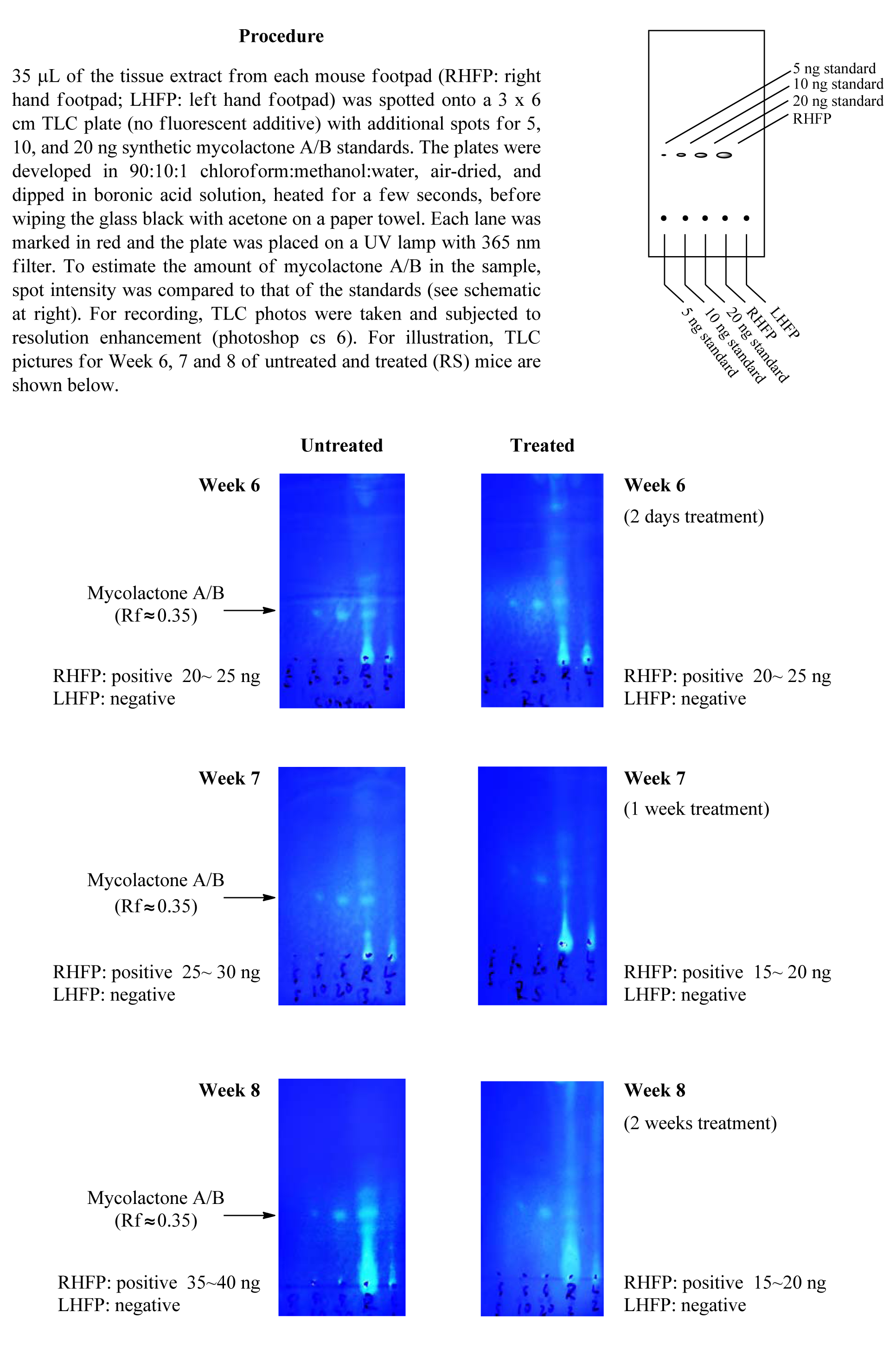

Supplement: Figure S1 — Schematic of fluorescent thin layer chromatography (f-TLC) procedure with examples. Top left) Explanation of the f-TLC method; Top right) Schematic of f-TLC layout. Center, Stained TLC plates of mouse footpads, untreated on left and treated (RS) on right, from top to bottom: 6, 7, and 8 weeks after infection. (TIF) [file pntd.0002618.s001.tif]
